# Discussions of the Twenty-Seventh Annual Meeting of Mississippi Valley Dental Association

**Published:** 1871-04

**Authors:** C. M. Wright


### Proceedings of Societies.

### DISCUSSIONS OF THE TWENTY-SEVENTH ANNUAL MEETING OF MISSISSIPPI VALLEY DENTAL ASSOCIATION.

REPORTED BY C. M. WRIGHT, D. D. S.

### FILLING TEETH WITH HEAVY AND LIGHT MALLETS.

Dr. J. Taft : No doubt is entertained by a large majority of our profession, of the value of the mallet in filling teeth. There is a diversity of opinion in regard to the kind, and manner of its use, and this diversity is no greater than the diversity in the circumstances of its use. The efficacy of the mallet is very much modified by the difference in the method of use. He would prefer the hand pressure, to irregular, slanting, unsteady blows from the mallet. An assistant should be able instantly to catch an indication from the operator. Dentists make the poorest malleters because they will let the eyes pass to the wrong point of the instrument. He has tried all the kinds of mallets in use, and finds a great variety of susceptibility among patients, in regard to the kind and weight of the mallet. One will bear a heavy lead mallet, while another will wince under it and feel the distribution of force as a jarring sensation, that they do not complain of under the light wooden mallet. Has never found a mallet yet that was not intolerable to some patients. He does not believe that any experiments on any machine will determine accurately the effects of the mallet on every patient.

Dr. J. G. Willis: Agrees with Dr. Taft about the difference in the sensibilities of patients to the forces of the mallet. Uses himself a small wooden mallet, and yet thinks that a great deal depends upon the assistant. Patients will bear uniform blows even if the momentum be greater than irregular blows. Nothing vexes an operator more than a poor malleter, and as filling teeth requires time, it also requires composure of the mind of the operator. Anything that gives assurance promotes success in filling teeth. He wants to depend on the regularity of the mallet blows upon his dam; for freedom from moisture, etc., to be in mental condition to make a good plug. Thinks the difference in mallets themselves not so important a matter. The same patients vary in their susceptibilities, under the same mallet and same force. Thinks the light mallet the most agreeable. In regard to the rapidity of the blows, stated that when two blows of exactly the same momentum are struck, the last one seems the hardest, because the impression of the first has not had sufficient time to diffuse itself and the parts to recover for the second blow.

Dr. F. H. Rehwinkle: Allows an interval of time between the blows. Thinks that much more depends on the assistant than on the kind or weight of mallet. Has experimented with the wooden, the steel, and the lead mallet in the same filling, without the knowledge of the patient, and invariably finds the patients flinch under the heavy mallet. Thinks lead mallets less disagreeable to the sense of sound than ivory or steel. For his use prefers a light lead mallet of from one to one and a half ounces.

Dr. J. Taft: Objects to "*time* strokes," and prefers a stroke *indicated* by the operator. Especially is this important in working about delicate walls of cavities. The malleter should always wait till the indication comes from the operator. For eight or nine years he used the lighter mallets with the double stroke, but for the last year has used the steel mallet almost exclusively, and with this he has but one blow. The heavy steel or lead mallets are too heavy for the double blow. With the lead or non-elastic mallet, the force is distributed largely throughout the surrounding tissues, while with the steel or the elastic mallet, the force is expended nearer the point of the instrument. Would prefer to confine the distribution of force to the gold itself, and not have it extend through the tooth, jaw and head.

Dr. J. W. Baxter: Condenses the gold with one stroke. Uses small points and a light mallet.

Dr. H. A. Smith: The same blow on a large pointed instrument, will not produce the same consolidating force that it will on a smaller instrument. Should think a large point would distribute force more quickly. The smaller the point and the lighter the elastic mallet, the more agreeable are the sensations to the patient. Has tried with pretty good results a leather mallet. Spoke of frequent periostitis from over malleting. A great deal of harm may be and is done by the mallet. There are many teeth lost by the mallet. Skill and caution in the use of this most useful instrument is very necessary to success.

Dr. W. H. Shadoan: Uses a cork between the teeth when malleting on a lower tooth, to prevent the vibrations of the jaw, the contraction of the muscles, and as a support for the jaw. Has experimented with half a dozen mallets from half an ounce to six ounces in weight. Has found the two ounce ivory or three ounce steel mallet the best for a majority of cases. For very loose teeth uses a half ounce mallet with considerable velocity. In some teeth uses the rapid blow with light mallet, and in others the heavier mallet and slower stroke.

Dr. P. G. C. Hunt: Uses a heavy mallet of lead. From observation has learned that there is very much less periostitis under the heavy than under the light and more elastic mallet. When he *presses down* his instrument on the gold, before the mallet blow, finds the patients better satisfied. Has never had serious cases or death of the pulp, or periodontitis produced by malleting.

Dr. McKellops: Has used ivory, steel and lead mallets. Likes lead because it makes less noise. Lead has the objection however of being cut up by the instrument handle, and dropping in small particles on the patients.

Dr. Berry: Thinks the handle of the mallet should be thin and springy.

Dr. Rawls: The lead mallet, like hand pressure, distributes the force and is not so painful to patients. The large point distributes force and drives the mass of gold, while the small point *splits*, and drives in a more direct line. Believes it quite important to press the instrument solidly against the gold before the stroke. Thinks more pain accompanies the use of the small than the large pointed instrument, on the principle of the distribution of force.

Dr. Keely: After experiments with mallets of from one to ten ounces, has settled on a six ounce lead, because his patients complain less with this. Considers it important to press the instrument against the gold before the force. Said he did not think that he could to-day fill a tooth by hand pressure, so dependent is he on the mallet. Advocates unhesitatingly the lead mallet.

Dr. Taft: The large pointed instrument pressed against the gold, and struck with a heavy mallet, permits a distribution of the force, that the fine point and a rapid light blow does not. The fine point and rapid blow consolidates the gold in the immediate vicinity of the point, and does not effect so much the mass. Does malleting produce periostitis? It is not so liable to occur as from hand pressure. The malleting is sometimes a remedy for periostitis. Related his own case, of a sore molar cured by the malleting of a filling some ten or twelve years ago, that had never troubled him since.

Dr. McKellops: Carries his gold to the desired point with pliers, and then frequently uses the plugger as he would in chasing, not pressing it against the gold. Uses the small point in preference to the large on heavy foil. In regard to periostitis, thinks it more liable to occur from the mallet, than from hand pressure force.

Dr. Morgan: Does not use the mallet universally. A tooth around the root of which there is inflammation, can be filled with less pain with the mallet than with hand force, because the force is not so generally distributed by the former. Any one can make a harder filling with the mallet.

Dr. Rawls: Explained that the sudden stroke of the mallet breaks up and drives away the globules of blood at the point of stasis, about the tooth in periostitis, and this is the reason malleting cures periostitis in the cases referred to. On the homœopathic principle *similia similabus curantur.* A blow will produce periostitis and a consequent stasis of the blood, and a blow may break up the trouble and cause the blood to circulate in a normal manner.

Dr. Taft: When there is only a simple stasis this theory may be correct, but when a bruised condition of the part presents, a blow may produce serious periostitis.

SECOND DAY.

### *Treatment of Soft Tissues of the Mouth.*

Dr. Taft: A large proportion of the diseases of the teeth, arise from an abnormal condition of the mucous membrane, and a vitiated state of the secretions. Every one in practice has given some attention to the more apparent diseases of the mouth. Tumors, abnormal growths and similar affections. Even these can be better understood and treated than at present, but there are other diseases more obscure that should receive our attention. One is that condition, that has not reached inflammation, but is an over stimulated condition of the mucous membrane, the result of some irritation. This causes an increase of the secretions and acidulates them, producing an active agent for the decay of the teeth. Sometimes the alimentary canal affords causes for this trouble, but frequently in the mouth itself, the mucous follicles are diseased, though not particularly sensitive, and throw out a secretion of an acid reaction. Whatever changes the normal condition of the mucous membrane, is liable to injure the teeth. The mucous membrane is affected frequently by local causes—deposits on the teeth, filthy condition of the mouth, foreign substances in the mouth, dental plates whether they fit well or badly, in short, anything that comes in constant contact with this membrane, making pressure, may produce this effect with the result named, to the teeth and general health. The thermal effects of certain plates are objectionable in some cases, such as non-conducting substances that do not allow a sufficient escape of the caloric of the mouth, even when no decomposition takes place in the plate itself. Even platinum can not be worn in some mouths, the simple contact of which causes trouble. In practice we should be prepared to substitute the proper material when these conditions are presented—what is best for this particular case should be our inquiry.

Dr. Watt: One condition of mouths we have frequently to observe and not attribute to other causes, is the "nursing sore mouth of women." Generally an impoverished condition of the blood accompanies this disease. The treatment of these cases is so affected by the constitutional conditions of the patient, that we are frequently too restricted in our treatment, *i. e.* we treat too topically. Sometimes these cases are treated with nitrate of silver or carbolic acid, every day, or every other day without benefit. The treatment is wrong. The blood must first be purified. Physicians used to say to the mother with this sore mouth, "you must wean your child and stop this drain, as the impoverished condition of your blood will not bear it." Thinks this in a large majority of cases unnecessary. The zymotic diseases are of this character, produced as they are by foreign substances in the blood, having less vitality than the blood. This virus must be got rid of, and if we can so adjust our remedies that they will kill this poison, and not affect the healthy part of the blood, we have the right treatment to begin with.

We want to rid the blood of the rubbish, and then begin to build—knows of nothing better than a mild course of arsenic. The water from springs that contain a trace of arsenic are invaluable in these cases. Doses of from 1–100 to 1–50th of a grain of arsenic are good. Mild topical applications of carbolic acid also may be employed. This treatment is fatal to the foreign organic matter in the blood, and the ulcers soon begin to grow less. Then we are ready to build. Without this constitutional treatment, the local treatment is too frequently only time misspent.

Dr. Roudebush: Has never seen a case of nursing sore mouth cured, even under arsenic treatment, unless the babe was weaned. Has used Fowler's solution, beginning with two drops and gradually increasing the dose to six.

Dr. Watt: In all these cases wants the mother or the preacher with the sore throat to proceed with their business, and he will treat the disease right on at the same time. Urges the mother to spare the babe the terrible misfortune of not nursing. Has never had a mother to wean her child for this cause.

Dr. Taft: Referred to a case of Dr. Watt's treatment, and said that the babes who were not weaned showed a marked improvement over those who by the advice of other physicians, at other times, had been weaned. This treatment is not only for the parent but is a benefit to the children.

Dr. Watt: The treatment usually begins with arsenic, and is followed by the phosphates of lime. Nourishing diet is the best material with which to build, good beefsteak, newly made cheese, etc.

Dr. Taylor: These little ulcers that appear in the mouth in some cases and at times seem to be epidemic. Referred to an article from Dr. J. B. Smith some years ago on this subject, published in the *Register*. Has noticed this same thing since that time. In these cases the ordinary local treatment of tinct. of Myrrh, Arnica, etc., seemed to be pernicious; increased the difficulty; produced burning sensations, etc. We must go back to the blood and purify it, restore the circulating fluid to its natural condition, and then rebuild. Has given arsenic as a tonic; this excites an appetite for the food that is desirable. The elements we want are found in food and in better quantities than in medicines proper. Has lost confidence in many of the preparations of phosphates.

Dr. Roudebush related a case of a lady with a babe three months old, to whom he gave chloroform and extracted teeth. After this she had sore mouth. He made a gold plate for her, but her mouth got worse. Made a platinum plate, still the mouth grew worse. A physician in the mean time had treated her with arsenic and the phosphates, the babe died and the patient got well immediately.

Dr. Watt: Does not regard Fowler's solution the desirable officinal preparation. It has to undergo decomposition before the arsenic is free to act. Has always had better results from arsenious acid, not combined with a base. Fowler's solution is the arsenite of potash.

Dr. Morgan spoke of the peculiar character of some springs in his neighborhood (Nashville, Tenn.) which have the desired effect in these cases. There are cases of sore mouth in females of from thirteen to twenty years of age, and in men, where the local treatment will not cure, and where the arsenical treatment is necessary. Has observed the epidemic character of this disease. In all these cases has not found local remedies that contain alcohol so beneficial. In his practice has therefore laid aside tinctures. Prefers decoctions. The decoction of white-oak bark is a valuable remedy.

Dr. Taft: There is a strong disposition to use local remedies in these cases. They may some times relieve but must not be relied upon. The chief reliance is upon systemic remedies, and that these may be rational we must learn the causes of disease. If poison in the blood be the cause, it should be neutralized or eradicated. Some poisons we can not eradicate, but we may control them to a certain extent. Malarious poisons may be removed, while usually syphalitic poison can only be controlled.

In building up we want those elements that enter into the formation of cells, the nitrogenous agents that make muscular tissues. First, however, we must find the enemy and then rout him.

THURSDAY, P. M.

### *Materials for Filling Teeth.*

Dr. Taft: Gold is of course the best filling known, though it has some serious objections. Its conductibility is one, and another, the difficulty of adapting it to the purposes for which we most need it. He spoke of the more easily manipulated and more easily adapted materials, amalgam, Hill's Stopping, os-artificial, and referred more especially to Guilloi's cement. This hardened slower, but when well protected for an hour or two from moisture, makes a very good filling. Hill's stopping and gold are all we can at present rely on. Gold is very variable; from one manufacture will be soft, tough and adhesive, while at another time, even from the same ingot, it works entirely different. Other metals show the same disposition. Steel, for instance, is in one condition brittle, and at another springy and tough. The method of the manipulation of gold foil to produce the best and equal results is not fully understood even by the manufacturers.

Dr. Morgan remarked that he had met with the same difference and had failed to learn even from the gold-beaters the causes. The question has been asked, at what thickness of gold can we get the maximum softness. Gold from different localities show different degrees of softness. The southern mines produce a paler but a softer gold. The gold from Georgia and South Carolina seems to be freer from iridium. He referred to his use of this new French cement, and thinks it very nearly the same material we call os-artificial. The setting of os-artificial can be affected by the temperature, and if mixed on a thick slab of glass, after taken from ice-water, will set *slower*.

Dr. H. A. Smith: Thinks that if gold is perfectly pure, it is the same in all its working conditions. The alloys affect the working qualities. By increasing or diminishing the alloys, the molecular mobility of the mass is effected. To obtain a homogenous mass, certain definite proportions of the metals must be used. French metallurgists have given this subject much attention. Thinks Hill's Stopping a better filling generally than os artificial, and would rather trust it than Guilloi's cement.

Dr. Taft remarked in regard to the thickness of the foil, his experience warranted him in thinking no gold under No. 4 valuable, that from No. 4 to No. 60 are the most desirable. His practice lately has been to make the body of the filling with No. 4 or 6, and to finish with the heavier foils, 30 and 60. A good deal depends upon the condition of the gold. If we could get the gold always just right, the heavier foils would then perhaps be better than the light for the whole. The advantage of using heavier foils on the surface is that a better finish is secured. He finishes by laying on the flat, smooth pieces, and welding perfectly about the edges, by letting the heavy gold overlap the thin wall, or rather to hug down on the friable edge and thus secure and protect it. This can be better done with No. 60 in his hands than any other gold. We can save more of the form of the tooth with this method and No 60 than with thin foils.

Dr. McKellops now confines himself entirely to heavy gold foil. Entirely ignores amalgam. Uses, of course, Hill's stopping and os-artificial, especially in children's teeth, but not for any class of permanent fillings. Knowledge of the use of heavy foils increases every day and hour his confidence. Thus the introduction of heavy foil has improved the quality of foils in the market. Does not think that we can get different quality of gold from the same ingot, but it may be from different meltings. Thinks he can accomplish anything with gold that any man can with amalgam, and that if he could hold an egg-shell steady he could fill it perfectly with heavy foil. Urged a fair trial by the profession of heavy foil. Showed some specimens of heavy foil and some fine quality of rolled foil. Rolled gold is much softer and much tougher than the beaten foil. No. 20 will make perfect cylinders. In filling approximal cavities thinks it safe to leave spaces between the fillings in opposite teeth sufficient to avoid friction of the fillings in mastication, and to avoid the conditions that occasion the decay. Right here he asked to digress and make the positive assertion that in our dental associations, where these subjects are talked over, we find the quintessence of knowledge—knowledge that we may never get from books or by our unaided efforts, though our solitary labor be ever so great.

Dr. Watt: It is a most unreasonable thing to expect that pure gold will be exactly the same, at all times, after its reduction to foil. If each *sheet* could be beaten with the same hammer, and alone, and the particles should yield in the same directions under the hammer, then perhaps, gold foils would all work alike. Rolling is certainly the most philosophical method of preparing gold for dental purposes. The fibrous condition is better preserved. When you strike the gold with the hammer the fibres are split.

Dr. Willis: Compared an unorganized profession to a regiment of soldiers straggling along without regard to rank or time. Every man in an organized profession, helps some. The heavy foils are introduced, and this excites the attention of the whole profession to the subject of good foil, and brings better work from the gold beaters, brings better instruments with which to manipulate the better gold, then the rubber dam comes forth, and all help to better results from our hands. Nothing is so annoying as to hear that this or that man is the "*best* operator in the country." The man who saves the largest proportion of the teeth he operates on is the best dentist, whether he does it with light or heavy, soft or adhesive foils.

There are physical laws against the use of very heavy foils—for the thicker the foil the harder it is—the more unyielding, etc.

An objection to Os-artificial and Guilloits' cement is, its disagreeable roughness when in contact with the tongue.

FRIDAY, P. M.

### *Microscopy of the Teeth.*

Dr. Taft: Detailed some experiments he had instituted in this direction. Referred more particularly to the anatomical structures of the teeth, rather than to the pathological conditions. Perhaps the profession generally are not aware of the great variety exhibited in the structures of the teeth. There is a general similarity, of course, as there is between men, but the variety is as great as the variety in men's faces. Showed some diagrams of about 3,000 diameters, exhibiting the tubuli of the dentine, in very great variety. Interglobular spaces are quite rare, only appearing in cases where there has been interrupted development. The spaces between the enamel and dentine vary in different specimens. The tubuli all have a general directian toward the periphery of the dentine, but vary largely in the regularity of their curves or courses, some running in straight lines, some in curves of various degrees. Sometimes their tubuli have branches. Sometimes they terminate in the granular layer between the dentine and enamel, and sometimes they end in fan shape terminations, being lost in the enamel itself. At other times the tubuli seem to pass through the enamel, even the cusp of a tooth. He also showed a diagram of a palatine molar root in sections, where on one side the tubul run into a line near the pulp canal, and on the other they seemed to run around the canal. Diagrams were also shown where the secondary dentine was exhibited in the pulp canal. In one the tubuli run through the secondary dentine. This internal deposit is very irregular in appearance. A question to be decided by future observations and experiments is: "where a sensitive part of a cavity presents, do the tubuli appear in great numbers, or fewer, etc.?"

Ordinary cementum has only the lacuna, little lakes with canalicula or spider legs, and is without the tubuli.

### FIFTH SUBJECT.

### *Dental Therapeutics.*

Dr. Rehwinkle: Spoke of the great need of more knowledge on this subject, of the demand for a special dental text book on the subject.

Dr. Osmond: In sensitive dentine, in those cases where the dentine seems to be almost gelatine, uses nitrate of silver in a saturated solution. After the application the places look like black decay, but the disease proceeds no further. He referred more particularly to those cases of sensitive dentine near the margins of the gums. Where this disease occurs there is a lack of calcarious substance, and a course of the phosphate treatment is necessary. Urges patients to stop the leakage of the natural phosphates by hard study and other exhaustive brain work, and to avoid the alkalies in food, salaratus in hot biscuits, etc. He makes saturated solution of the local remedy, nitrate of silver, by taking a piece of cotton wound round a little wooden toothpick, and with parafine, rubs it on the stick of nitrate of silver, and then applies carefully. In persons of strumous diathesis employs the phosphates. Prefers Churchill's preparations of the phosphate of lime and hypophosphate of soda in equal parts.

In ulcerated teeth where the sac appears, or periodontitis—a pyogenous membrane, a chronic inflammation exists—we want to get up an acute form of inflammation. After treating systemically, he pumps the iodine and carbolic acid into the sac. Where no external opening exists, he leaves the canal open after injection.

Dr. Watt: Agrees with Dr. Osmond in regard to the nitrate of silver. It combines with the albuminous tissues, and to the extent it combines death ensues. By applying it in this way there is but very little death; then by building up the system the result is good. Objects to the chloride of zinc in these cases. It causes pain. The blackened part forms a perfect covering while the dentine underneath is repairing.

Dr. Osmond: Applies nitrate of silver in sensitive cavities, when the pulp is not very far off, with better results than any other remedy.

Dr. Willis: Wished to enter his protest against the practice of injecting strong carbolic acid into an ulcer or sac at the end of a root, because it gets up such an inflammation. As favorable results can be accomplished with dilute carbolic acid, though it seems to require a longer time. Objects to violent means in therapeutic treatment. The death of the pulp is the universal cause of the abscess at the point of a root. We must stop up the root to keep out the gas formed by the decomposition of animal matter. Apply a pledget of cotton saturated with dilute carbolic acid. Sometimes the tubuli are filled with gas, and when the pulp canal is filled, this gas in the tubuli must escape through the canalicula of the cementum. Periostitis results from this, and the pus will escape around the neck of the tooth. He does not inject medicines at all, but applies it on cotton thread. Mixes carbolic acid with glycerine.

To prove that decomposition goes on in the tubuli, asked if when a plug is removed from the crown, even when the root or roots are hermetically sealed, an intolerable odor is not usually noticed?

Dr. Roudebush has treated teeth for sixteen years, and must acknowledge with poor success. Does not think he saves one tooth in ten. Honestly acknowledges that he has never saved or restored to perfect health a tooth with a sac at the root. They appear cured, but the disease breaks out again in a year or two. Objects to nitrate of silver on account of the discoloration, in front teeth. Has used carbolic acid, arsenic, and even cobolt for sensitive dentine, with pretty good success.

Dr. Willis: In former practice among surgeons there has been a prejudice against admitting air into abscesses. This care is of importance, and it is as necessary in the treatment of abscesses or roots as anywhere else. The oxygen of the air is not objectionable, but the irritating particles of animal matter afloat in common air are. Prof. Lister, in opening an abscess, always used to dip his lancet in carbolic acid, cover the abscess with carbolic acid, and then lay over it a piece of cotton or linen cloth to prevent the entrance of these irritating particles.

Dr. Taft: All the circumstances must be taken into consideration in the treatment of these cases, if we would gain success. If they are not understood and regarded you will fail in ninety-nine cases in a hundred. What he means by a success after this treatment is that a tooth shall be made comfortable and useful for the longest practical period. When there is a good degree of vitality in the cementum and dentine, the trouble referred to by Dr. Willis will not occur. In cases where it does we can surely help nature to carry off the debris. Has not found so great difference between carbolic acid and creosote in the treatment of these cases; both form an insoluble compound with the albumen and fibrin, and both are stimulants.

Dr. Morgan: Very much of our success in such cases depends on the systemic condition of the patient. Thinks it unadvisable to attempt the cure of abscesses in the molars of a patient of strumous diathesis or in scrofulous patients. Treats through the pulp canal or alveolus, and frequently makes an opening to the point to be treated. Sometimes there appears to be a calcarious deposit on the point of a root, but analysis has found it composed entirely of animal matter. Sometimes simply cutting this away, or scraping the point of the root, or cutting away a little of the cementum and dentine at this point, is all the treatment necessary. Uses creosote in full strength frequently.

Dr. Rehwinkle thinks too much stress is usually laid upon the mechanical treatment. When we go to work to save we go to work and kill by filling root and crown. We must treat intelligently, and must even put our patient to bed, if necessary to save a tooth. They sometimes require blue mass and saline cathartics.

Has heard of operators who fill with wonderful skill up into the tortuous and crooked canals of a pulp, and go even beyond that and make a rivet-head on their gold on the other end of the apex, and then he has felt that he has mistaken his calling, for he desires with becoming modesty to acknowledge that he can't do this.

